# Evidence of niche differentiation for two sympatric vulture species in the Southeastern United States

**DOI:** 10.1186/s40462-019-0179-z

**Published:** 2019-10-30

**Authors:** Amanda E. Holland, Michael E. Byrne, Jeffrey Hepinstall-Cymerman, A. Lawrence Bryan, Travis L. DeVault, Olin E. Rhodes, James C. Beasley

**Affiliations:** 1 Warnell School of Forestry & Natural Resources, University of Georgia, Athens, GA USA; 2 Savannah River Ecology Laboratory, University of Georgia, Aiken, SC USA; 3 Present address: School of Natural Resources, University of Missouri, Columbia, MO USA; 4 USDA/APHIS/WS National Wildlife Research Center, Sandusky, OH USA; 5 Odum School of Ecology, University of Georgia, Athens, GA USA

**Keywords:** Carrion, Competition, Landfills, Resource selection, Roost habitat, Savannah River site

## Abstract

**Background:**

As obligate scavengers utilizing similar habitats, interspecific competition undoubtedly occurs between resident black (*Coragyps atratus*) and turkey (*Cathartes aura)* vultures. In the interest of exploring how sympatric species coexist through habitat segregation, we examined resource selection of resident black and turkey vultures in the southeastern United States (US) for evidence of niche differentiation.

**Methods:**

Using fine-scale movement data, we assessed interspecific seasonal differences in monthly roost reuse frequency and roost site fidelity, as well as monthly flight, roost, and diurnal rest site resource selection based on > 2.8 million locations of 9 black vultures and 9 turkey vultures tracked from September 2013 to August 2015 using Groupe Spécial Mobile/Global Positioning System (GSM/GPS) transmitters.

**Results:**

Black vultures generally exhibited greater roost fidelity as well as a greater maximum number of nights spent at a single roost than turkey vultures. Patterns of flight, roost, and resting habitat selection within the home range varied monthly as well as between species, providing evidence for habitat segregation and niche differentiation by sympatric vultures. In particular, our results indicate the importance of wooded wetlands for resting and roosting locations for both species, and revealed clear differences in the use of forested habitats between species during flight, resting, and roosting behavioral states.

**Conclusions:**

By examining differences in resource selection and spatial ecology of black and turkey vultures across a range of behaviors, this study demonstrates mechanisms of niche differentiation in these ecologically similar species, and enhances potential for conservation and informed management of this important group of birds.

## Introduction

Competition between sympatric resident, non-migratory species occurs throughout the annual cycle when resource requirements are similar; however, niche differentiation reduces competition and allows for resource partitioning and coexistence [1–3]. Interspecific competition is particularly relevant for vultures (i.e., obligate scavengers) as both Old and New World species are specifically adapted to relying on carrion as a primary food resource [4]. As the only known terrestrial obligate scavengers, vultures collectively possess numerous evolutionary advantages for detecting and consuming carrion over facultative scavengers, including broad wings for soaring, excellent vision, and highly acidic stomachs [5]. In areas where multiple vulture species coexist, niche differentiation among vultures is further demonstrated through variation in morphological characteristics such as body size, skull size, beak strength, and mandible metrics, characteristics relating to feeding strategies (e.g., “rippers” with wide skulls and strong beaks, and “scrapers” and “gulpers” with narrower skulls and weaker beaks) [4]. However, in many areas, body sizes and feeding strategies are not notably distinct between coexisting vulture species, and niche differentiation can occur through differences among species in behavior [6–9] or physiology [10]. Similarly, many species may further minimize interspecific competition to facilitate spatial and temporal niche separation through habitat segregation [11], although this aspect of vulture coexistence has been largely understudied.

In areas of sympatry, interspecific competition undoubtedly occurs between black and turkey vultures as both species are diurnal, obligate scavengers utilizing similar roosting [12–14] and nesting habitats [15, 16]. Turkey vultures primarily forage solitarily or in pairs and have an enhanced sense of smell [10, 15], allowing them to exploit carrion in areas where visual detection is limited, such as in forests with dense canopy cover [9]. Conversely, black vultures have a minimal sense of smell, forage with conspecifics, maintain large social groups, and respond to visual cues of other scavenging birds for improved carcass detection [16, 17]. Black vultures are known to enhance foraging efficiency by information sharing and local enhancement (i.e., when an individual vulture is drawn to a carcass as signaled by the presence of other scavenging birds) [18]. Groups of black vultures are generally aggressive and are able to displace individual turkey vultures from carcasses [16, 17]. For sympatric vultures in the southeastern United States (US), movement activities and space use also have been shown to differ, with turkey vultures spending more time in flight and traversing larger ranges than black vultures [8, 9].

Although black and turkey vulture ranges overlap extensively, and both species are relatively common and abundant throughout their ranges, there are few studies elucidating the ecology and resource selection for these species. Moreover, inferences from prior assessments of home range and resource selection have been limited by the capabilities of tracking technology – i.e. physical observation [19], Very High Frequency (VHF) radio telemetry [20, 21], and fixed-wing aerial radio telemetry [22, 23]. Although these previous studies are useful, more recent advancements in Global Positioning System (GPS) tracking technologies allow for high-resolution location sampling and have greatly improved our understanding of how vultures partition resource use through both space and time [8, 9, 24, 25]. Such data, when coupled with high-resolution land cover data, facilitate examination of resource selection for evidence of resource partitioning at finer spatio-temporal resolutions than previously possible, including characteristics of roost selection and measures of roost reuse and roost site fidelity, which are poorly understood. To date, no prior studies have examined variation in black and turkey vulture resource selection across finer temporal scales (e.g., monthly) [26].

The goal of this study was to explore evidence of niche differentiation in sympatric black and turkey vultures in the southeastern US using fine-scale movement data to elucidate spatial and temporal differences in resource selection throughout the annual cycle as a function of species and vulture behavioral state (i.e. flying, resting, and roosting). To achieve this goal, our specific objectives were to: 1) quantify monthly differences in the reuse frequency and site fidelity of black and turkey vultures to evening roost sites; and 2) document differences in monthly selection of habitat attributes associated with flight, evening roost, and diurnal resting sites between black vultures and turkey vultures. We considered distinctions in selection of habitat attributes, uncovered by these data, to demonstrate mechanisms of niche partitioning and facilitation of coexistence among these ecologically similar species. We predicted evening roost habitat selection, roost reuse frequency, and roost site fidelity would vary throughout the year and between species. Specifically, due to their more gregarious social structure, we expected more frequent reuse of evening roosts by black vultures, and that evening roosts would be composed of greater proportions of forested habitat in winter months for both species due to increased thermal cover needs. Furthermore, we expected monthly resource selection to vary between species and between flight and resting behaviors. Because nesting activities generally occur in areas of low-disturbance [15, 16], we hypothesized both species would increase use of undisturbed (low human-impact) areas during breeding season months. Considering morphological differences and behavioral differences in carcass detection between species during flight, we hypothesized turkey vultures would use greater proportions of forested habitats and black vultures would use greater proportions of open landscapes, including roads, agricultural areas, and other areas of human disturbance (e.g., urban development, landfills, etc. [4, 11, 15, 16]).

## Methods

### Study area

**Fig. 1:**
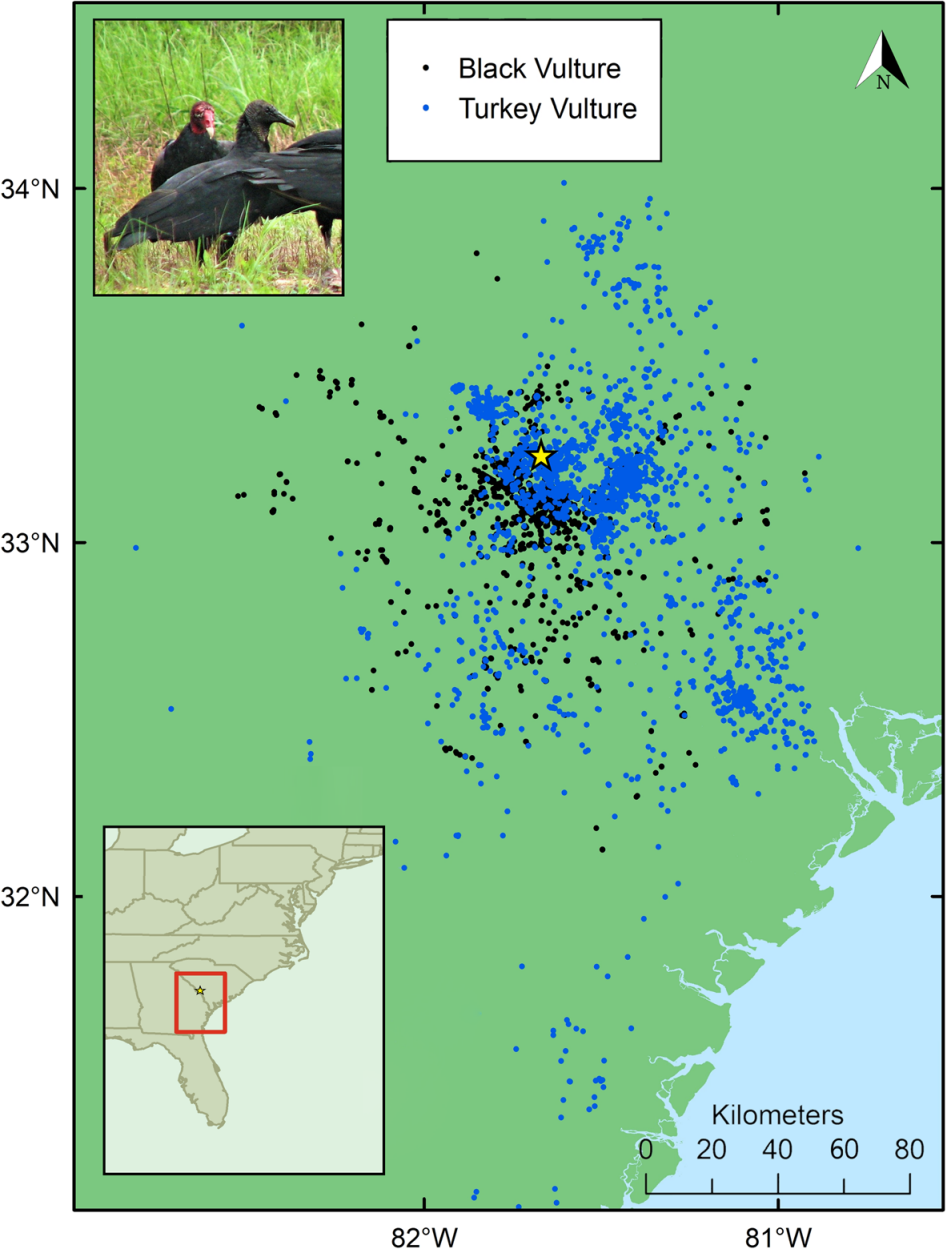
Roost locations for 9 black vultures (black dots) and 9 turkey vultures (blue dots) showing distribution of vultures within the study area central to the Savannah River Site (star) in the southeastern United States. Inset: image of black and turkey vultures foraging together (photo by Jim Beasley)

This research was conducted at the Savannah River Site (SRS), located along the border of Georgia and South Carolina in the southeastern US (Fig. 1). Populations of black and turkey vultures are abundant on the SRS which provides important roosting, nesting, and foraging habitat for both species [22, 23]. The SRS is a 780-km^2^, limited-access nuclear research facility owned and operated by the US Department of Energy (DOE). Much of SRS is relatively undisturbed by DOE activities and is primarily forested. The SRS is composed of planted pine forests managed by the US Forest Service, bottomland hardwood, wetland, and various industrial areas including five decommissioned nuclear reactors, radioactive materials processing plants, and landfills [27]. The SRS is located in the upper Atlantic Coastal Plain, which has minimal topographic variation (elevation ranges < 30 m to 115 m above sea level [27]). The composition of largely undisturbed natural areas and pockets of human-use industrial areas at the SRS make this site an ideal location in which to study resident vulture resource selection.

### Vulture Trapping & Handling

During summer 2013 and spring 2014, we captured 295 black and turkey vultures on the SRS. Of these, we fitted 13 adult black vultures and 14 adult turkey vultures with solar-powered 70-g Groupe Spécial Mobile/Global Positioning System (GSM/GPS) transmitters [8, 28]. Across multiple trapping events, only one vulture from each trapping effort received a GPS transmitter such that tracked individuals were not likely to be from the same foraging group. GPS locations were recorded at variable intervals with diurnal collection rate being highest at mid-day (1 min - 120 min) and lowest in the evenings (30 min - 240 min), depending on transmitter battery voltage [29]. Vultures were handled in accordance with the University of Georgia Animal Care and Use Protocol No. A2013 02–004-Y2-A2. All trapping was conducted during the non-migration season to target resident vultures. All vultures were affixed with numbered patagial tags for individual identification, measured for standard morphological characteristics, and aged (adult or juvenile) based on coloration and wrinkling of the head [15, 16]. Given that black and turkey vultures are sexually monomorphic [15, 16], it was not possible to control for balanced ratios of male and female black and turkey vultures from among those randomly selected to receive GPS tracking devices. Feather and blood from a leg or brachial vein was collected for use in sex determination via sex-specific deoxyribonucleic acid (DNA) markers. All genetic analyses were conducted at the Savannah River Ecology Laboratory in Aiken, South Carolina, as described in Holland et al. [8].

### Roost Fidelity and reuse

Prior to analyses, we filtered locational data to remove outliers including fixes with altitudes > 12,000 m, as well as missed fixes, 2D fixes and fixes with negative altitudes. We quantified monthly roost site fidelity and reuse using location data collected from August 2013–June 2015. We used the total number of unique roost sites used each month as a measure of roost fidelity, and calculated the maximum number of nights each month spent using a single roost as a measure of roost reuse. To identify evening roosts, we extracted the average spatial location from among telemetry fixes received after sunset and before sunrise. All locations were buffered by 75 m to incorporate location error (±23 m [29]). Neighboring roosts within 100 m were merged using ArcMap GIS (Geographic Information System) software version 10.1 [30], incorporating habitats between roosts to form a single roost and remove distinctions between locations that may be proximally considered the same roost as used by a vulture. To model monthly variation in roost metrics, we used generalized additive mixed models (GAMM [31]) using the mgcv package [32] in R [33] and specifying a Poisson distribution to fit cyclical spline functions to roost fidelity and reuse for each species, with vulture ID considered a random effect to account for individual variation in roost behavior.

### Monthly resource selection

We contrasted use of different habitat attributes each month for black and turkey vulture evening roost locations, flight locations, and diurnal resting locations separately relative to availability within each individual’s monthly 100% home range. Home ranges were delineated from utilization distributions (UDs) using the dynamic Brownian bridge movement model from filtered location data of black vultures and turkey vultures monitored from 1 September 2013 to 31 August 2015 (See Holland et al. [8] for details of home range models). Resource use was defined from filtered raw location data of vultures, and movement states (flight, resting, or roosting) were determined for each location based on movement speed and time of day. Field tests of the GSM/GPS transmitters revealed that reported instantaneous speed estimates were a useful indicator of movement activity [29]; thus, we categorized all diurnal locations reporting speeds ≥1 knot (~ 0.5 m/s) as “flight,” and all others “resting”. We delineated evening roost locations as described in the previous section. Once characterized, all locations were buffered by 75 m to incorporate location error (±23 m [29]).

To analyze differences in monthly resource selection by species in flight, resting, and roosting behaviors, we selected habitat attributes expected a priori to be important in providing vulture roosting, nesting, and foraging resources. We included distances to roads as a predictor for carrion resources because carrion is typically ephemeral and difficult to predict spatially and temporally, except along roads due to roadkill (but see Hill et al. [34, 35]). While different types of roads may elicit different responses from these species, we limited our analysis to distances from primary roads, where speeds are typically higher and the risk of wildlife collisions may be greatest. In addition, we used distance to landfills because vultures commonly use landfills as they provide relatively reliable foraging opportunities year-round [36, 37]. We also included land cover characteristics distinguished by canopy openness (i.e., open terrain such as crops and grasslands vs. closed canopy terrain such as forests and wooded wetlands). Furthermore, roost selection has been shown to be influenced by human disturbance [20, 38]; thus, we included habitat types (described below) with varying levels of human disturbance as an attribute expected to influence vulture spatial ecology.

Within buffered locations (flight, resting and roosting), we quantified the proportion of each habitat type (see Additional file 1: Table S1). We used six habitat types distinguished by ground-visibility and relative level of human disturbance including water (open water and emergent herbaceous wetland), wooded wetlands, forest (deciduous, evergreen and mixed), open developed (cultivated crops and hay/pasture), open undeveloped (rock/clay/sand, barren land), and developed/urban (low, medium and high intensity) derived from 30 m resolution land cover data from the 2011 National Land Cover Database [39]. Additionally, proximity to roads (derived from USA Major Roads map layer [40]) and landfills (derived from active solid waste landfill locations for Georgia [41], South Carolina [42], Florida [43], and North Carolina [44]) were binned into three Euclidean distance classes of 0-500 m, > 500–5000 m, and > 5000 m (proximal, medial, and distal, respectively).

Resource selection was determined by ratios of proportional use of each habitat type to its availability within the monthly 100% home range. Resource selection ratios (*ln* (rf)) were calculated for each bird, behavioral state, year, month, and habitat following the method of Rivers et al. [45] where:

1 $$ {\displaystyle \begin{array}{c}{Y}_{bird, sta te, year, month, habitat}=\\ {}\mathit{\ln}\left({rf}_{bird, sta te, year, month, habitat}\right)=\\ {}\mathit{\ln}\left(\frac{\mathrm{Proportion}\ {\mathrm{Use}}_{bird, sta te, year, month, habitat}}{{\mathrm{Proportion}\ \mathrm{Available}}_{bird, sta\mathrm{t}e, year, month, habitat}}\right)\end{array}} $$

For months where a resource was available but not used (i.e. proportion use = 0%), we replaced zero values with 1e^− 100^ to constrain the output of the equation to real numbers. We modeled monthly variation in selection for each habitat type by using GAMMs to fit a cyclical spline function to monthly selection ratios with vulture ID entered as a random effect term. To assess the support for monthly variation in habitat selection we compared the Akaike Information Criterion (AIC) values [46] of the GAMM model for each habitat to an intercept-only mixed effects model that held selection constant across months. We considered monthly variation supported when the AIC value of the GAMM was > 2 units below the intercept-only model. Models were run independently for each species and habitat type using the ‘mgcv’ package in R [32]. It is important to note that proportions of habitat availability were not relative to all other habitats in this analysis, but rather relative to actual availability within the 100% home ranges, a type III design according to Manley et al. [47]. For example, habitats within distances proximal to roads or landfills could also be composed of various proportions of forest, developed/open, wooded wetland, or other possible habitat combinations included in this analysis. Therefore, selection ratios greater than zero (*ln* (rf) > 0) indicate the habitat type is used in greater proportion than its availability within the home range, i.e., “selected”, but does not necessarily imply avoidance of other habitat types.

## Results

Twenty-seven vultures (13 black vultures – 5 males and 4 females, and 14 turkey vultures – 6 males and 3 females) were trapped, tagged, and affixed with GSM/GPS tracking devices resulting in 2,823,627 locations collected among all vultures from 13 June 2013 to 31 August 2015. Due to small sample sizes, data for males and females were combined for all analyses. Transmitters often collected several hundred locations per day, although the relocation frequency varied depending on time of day, extent of cloud cover, battery charge, and time of year [29]. Non-migration/residency was verified by assessing net-squared displacement for each vulture [8] from evening roost sites. After excluding data collected from vultures whose movement did not span a full month (i.e. partial month tracks) and months wherein vultures exhibited migratory movements (see Holland et al. [8]), we estimated 322 monthly 100% home ranges from 9 black vultures and 9 turkey vultures (*n* = 150 and 170, respectively). We did not examine differences in resource selection by migrant and resident individuals due to the limited number of individuals exhibiting migratory behaviors (two turkey vultures, one of which only migrated in the first winter).

### Roost Fidelity and reuse

**Fig. 2:**
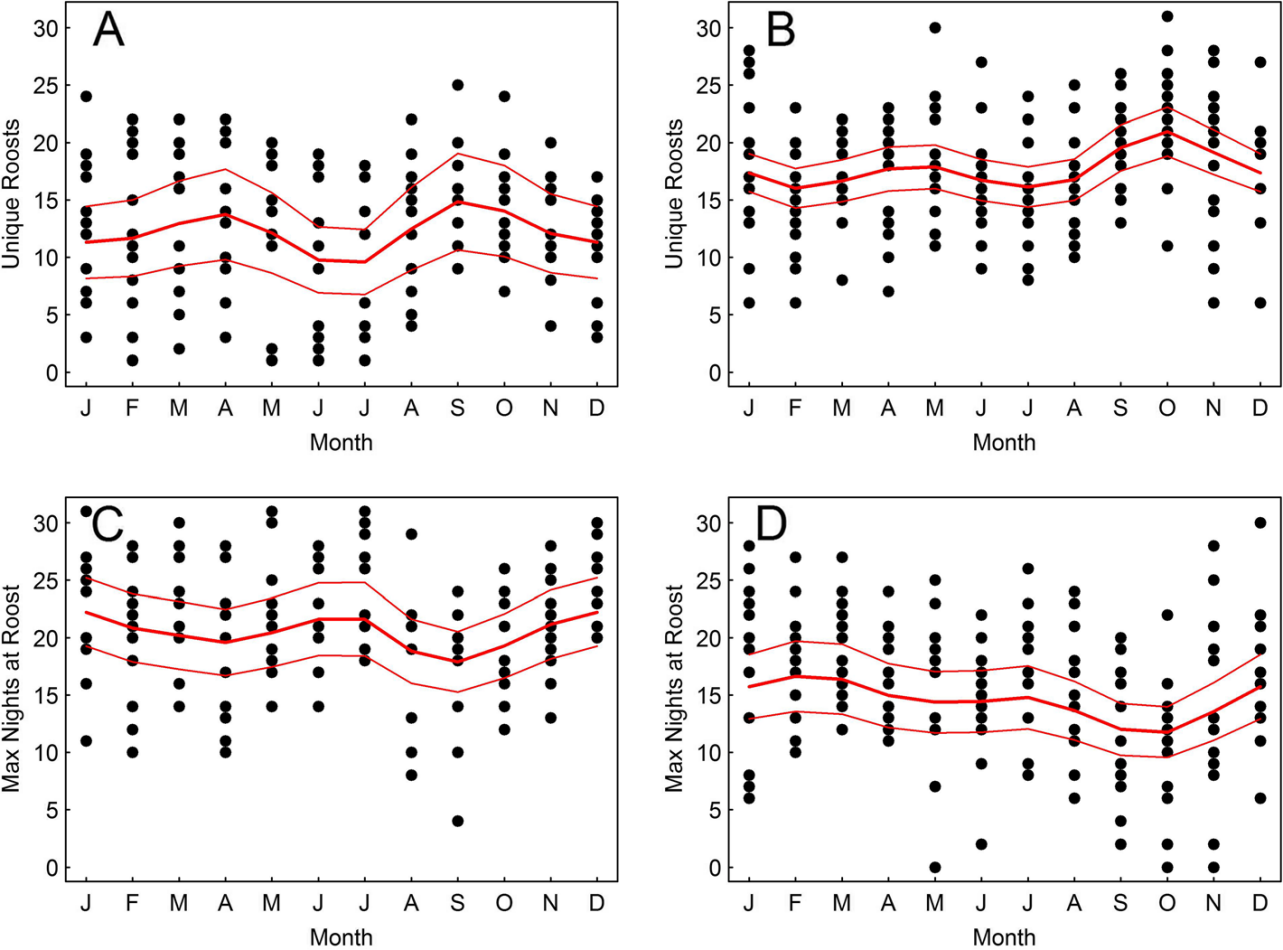
Number of unique roosts used by a bird during a month for 9 black vultures (**a**) and 9 turkey vultures (**b**) and maximum number of nights spent at a single roost for black vultures (**c**) and turkey vultures (**d**) monitored with GPS transmitters from September 2013 to August 2015. Fitted Poisson GAMM with 95% confidence intervals plotted in red lines

Although there was considerable interspecific variation, in general black vultures exhibited greater roost fidelity than turkey vultures. Overall, black vultures used fewer unique roosts per month (mean ± SD: 12 ± 6) than turkey vultures (18 ± 5; *t* = − 8.26, df = 299, *p* < 0.005), and black vultures spent more nights per month at a single roost (21 ± 5.5) than turkey vultures (15 ± 5.8; *t* = 9.04, df = 325, *p* < 0.005). Both species exhibited seasonal variation in roost site fidelity. Black vultures showed increased fidelity during the summer months of June and July, with decreased fidelity during spring and fall (Fig. 2). Turkey vulture roost fidelity decreased considerably in the fall, being lowest in October (Fig. 2).

### Vulture monthly resource selection

#### Evening roost selection

**Fig. 3:**
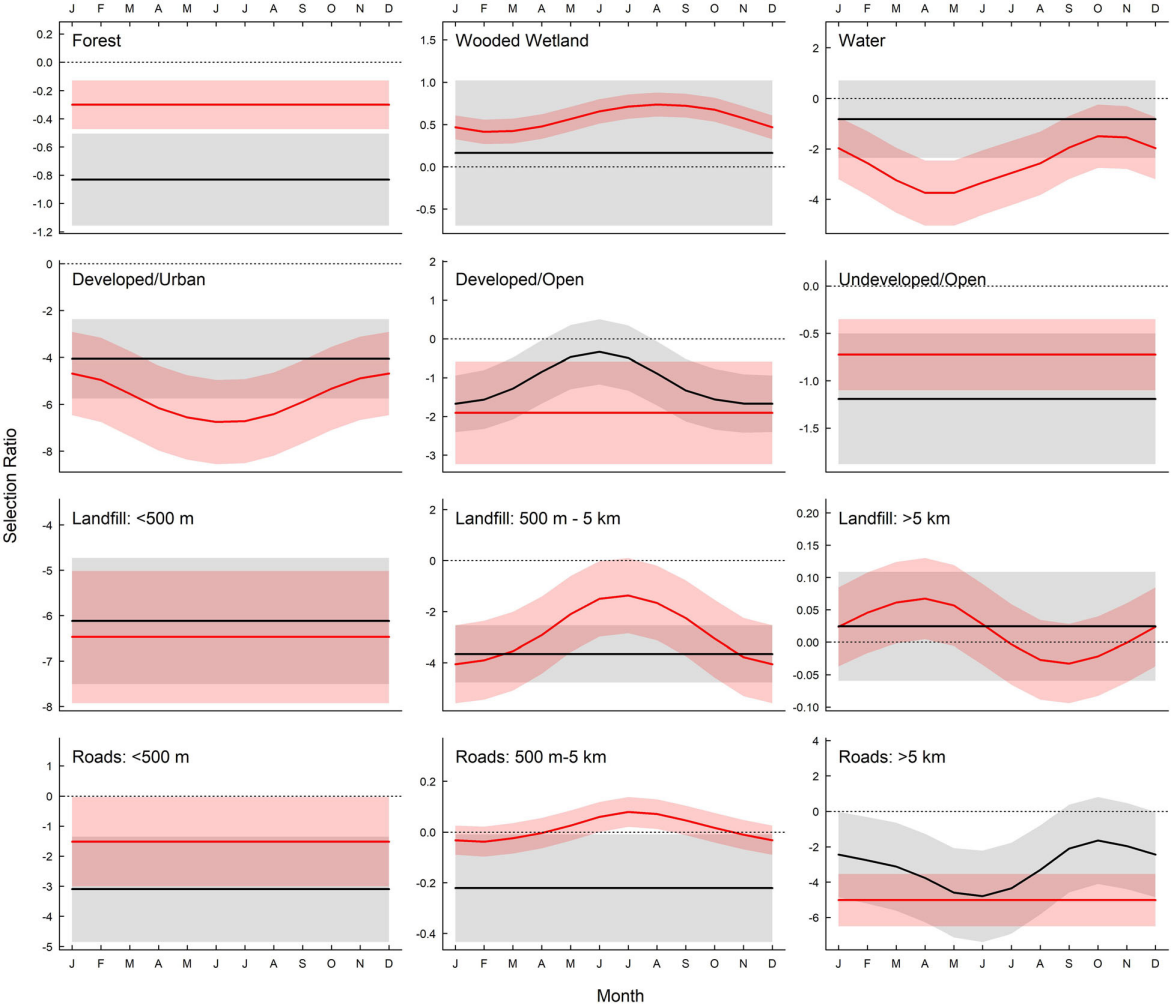
Mean (±SE) selection ratios (*ln* (rf)) modeled with GAMMs by month for evening roosts for 12 variables within 100% home ranges, calculated from locations of 9 black vulture (black) and 9 turkey vultures (red) tracked via GPS transmitters from September 2013 to August 2015. Straight lines indicate no support for effect of month; wavy lines indicate models including month as a cyclical smoothing term were > 2 AIC points lower than a model without the term. Shaded areas represent 95% confidence intervals. Selection ratios greater than zero (shown by dotted line) indicate habitat type was used in greater proportion compared to availability

Patterns of positive or negative roost site selection were broadly similar between species for most habitat types, although turkey vultures were more likely to exhibit monthly variation in selection ratios (Fig. 3; Additional file 2: Table S2). Both species avoided evening roost sites within forested habitat, with black vultures showing stronger avoidance (− 0.87 ± 1.22) than turkey vultures (− 0.32 ± 0.5). There was notable interspecific variation in selection of roosts in wooded wetlands. Turkey vultures exhibited positive selection for roosting in wooded wetlands in all months, with strongest selection observed from July to September (0.71 ± 0.44) as compared to the rest of the year (0.55 ± 0.40). In contrast, black vulture roost site selection in wooded wetlands were used according to availability (0.01 ± 2.53). Distance to landfills did not appear to be a positive driver of vulture roost site selection with both species avoiding roosts adjacent to landfills (turkey vultures = − 6.55 ± 3.89; black vultures = − 6.56 ± 4.33). Although selection ratios of roosts within distances medial to landfills were negative for both species, avoidance appeared to be greatest during winter months for turkey vultures (November – February; − 3.57 ± 5.06) compared to other months of the year (March – October; − 2.09 ± 4.27). Both species avoided using roosts proximate to roads (turkey vultures = − 1.30 ± 3.66; black vultures = − 3.44 ± 5.24). Black vultures avoided medial distances to roads in all months (− 0.27 ± 0.50), while turkey vultures exhibited positive selection during July and September (0.08 ± 0.14) compared to the rest of the year (− 0.02 ± 0.20).

#### Flight selection

**Fig. 4:**
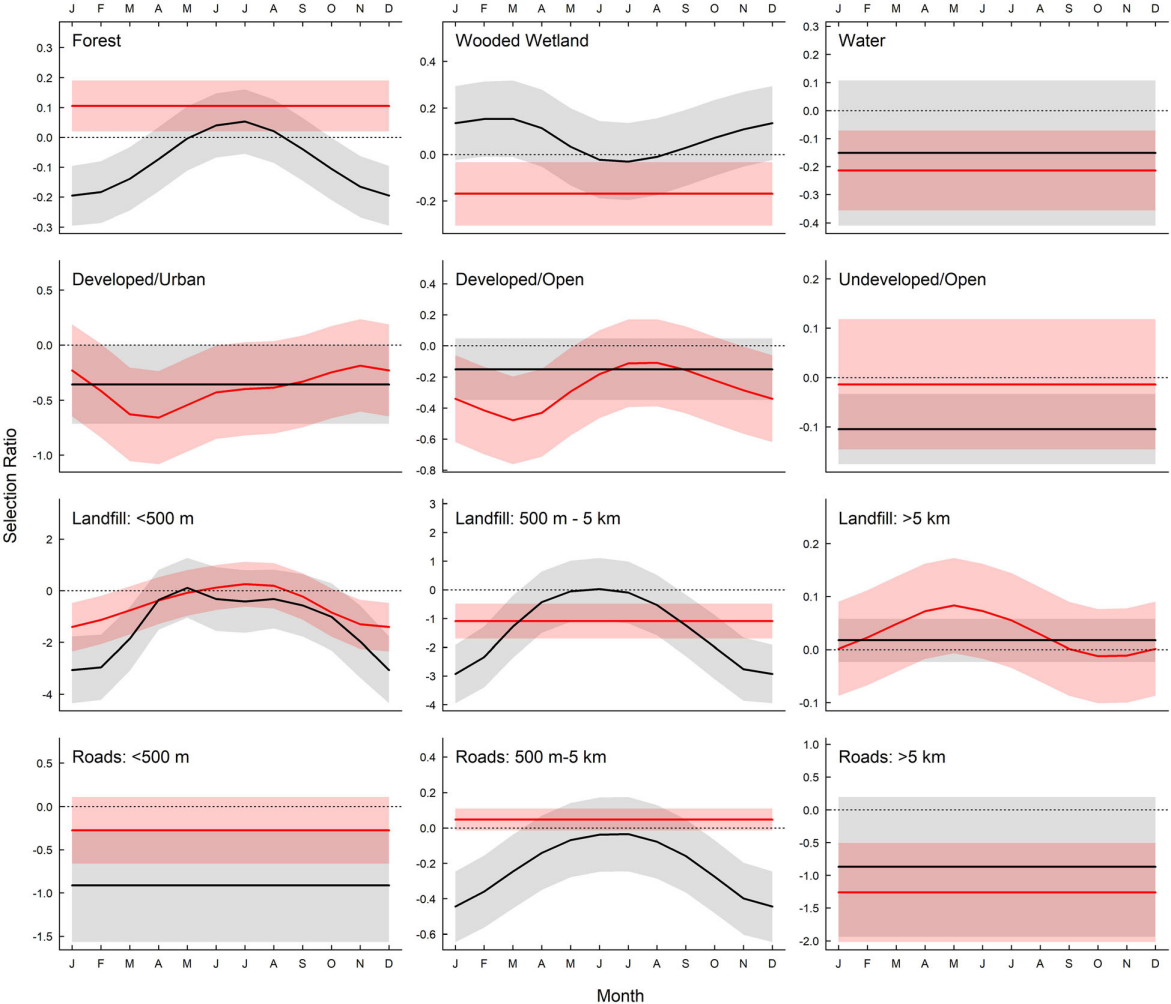
Mean (±SE) selection ratios (*ln* (rf)) modeled with GAMMs by month for flight for 12 variables within 100% home ranges, calculated from locations of 9 black vulture (black) and 9 turkey vultures (red) tracked via GPS transmitters from September 2013 to August 2015. Straight lines indicate no support for effect of month; wavy lines indicate models including month as a cyclical smoothing term were > 2 AIC points lower than a model without the term. Shaded areas represent 95% confidence intervals. Selection ratios greater than zero (shown by dotted line) indicate habitat type was used in greater proportion compared to availability

When selecting habitats during flight, models indicated several important interspecific differences (Fig. 4, Additional file 3: Table S3). Notably, turkey vultures selected forest habitat consistently in all months (0.09 ± 0.18), whereas black vultures avoided forest during cold-weather months (November – March; − 0.21 ± 0.25) and selected for forest during June – August (0.02 ± 0.21). However, black vultures selected for wooded wetlands across most months (0.06 ± 0.35) whereas turkey vultures consistently showed avoidance of wooded wetlands when in flight (− 0.16 ± 0.29). We also found limited evidence of interspecific differences in use of water, developed (developed/urban, developed/open), and undeveloped/open habitats with both species consistently avoiding these three habitat types when in flight. Temporal variability in selection of habitats proximal to landfills was evident as both species avoided flying proximal to landfills during colder months (November – February; turkey vultures = − 1.22 ± 3.51, black vultures = − 2.89 ± 3.89), and there was some evidence of selection of landfills when in flight during spring by black vultures (0.70 ± 0.95) and July–September by turkey vultures (0.55 ± 1.96). We also observed some evidence of interspecific differences in flight selection of habitats distal to landfills, although patterns of selection varied among months. Turkey vultures exhibited selection for areas medial to roads (0.04 ± 0.14), while black vultures clearly avoided flight in these areas during colder months (− 0.46 ± 0.56). Both species avoided flying in areas proximal (turkey vultures = − 0.25 ± 0.95; black vultures = − 0.92 ± 2.32) and distal to roads (turkey vultures = − 1.29 ± 2.09; black vultures = − 0.55 ± 2.40) across all months.

#### Diurnal resting selection

**Fig. 5:**
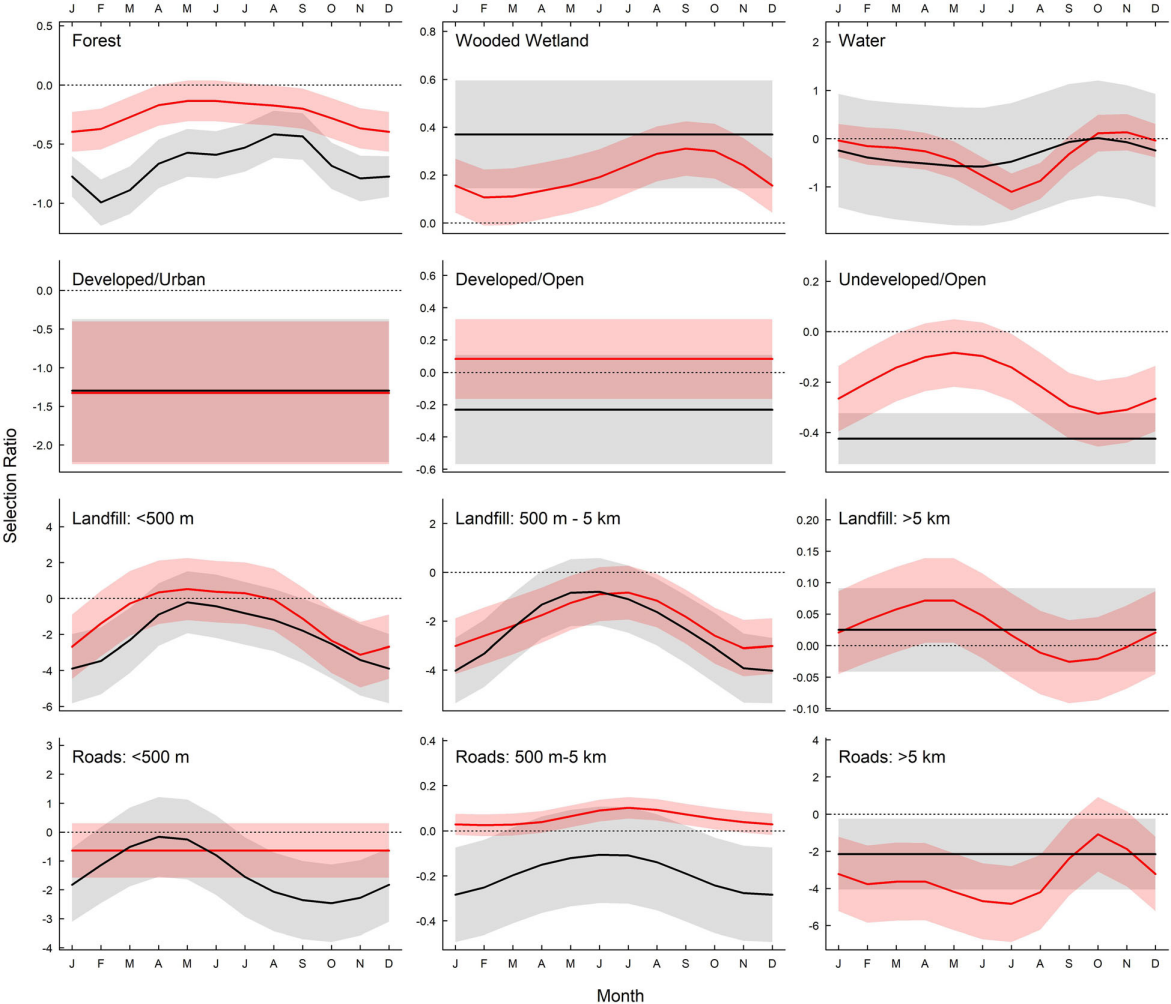
Mean (±SE) selection ratios (*ln* (rf)) modeled with GAMMs by month for diurnal rest for 12 variables within 100% home ranges, calculated from locations of 9 black vulture (black) and 9 turkey vultures (red) tracked via GPS transmitters from September 2013 to August 2015. Straight lines indicate no support for effect of month; wavy lines indicate models including month as a cyclical smoothing term were > 2 AIC points lower than a model without the term. Shaded areas represent 95% confidence intervals. Selection ratios greater than zero (shown by dotted line) indicate habitat type was used in greater proportion compared to availability

Mixed model analyses for vultures showed support for effects of species and month on diurnal rest site selection within most habitats within their 100% home ranges (Fig. 5, Additional file 4: Table S4). Both species showed monthly variation in avoidance of diurnal rest sites in forest. Black vultures consistently selected wooded wetlands to use for diurnal resting sites (0.32 ± 0.52) whereas selection varied by month for turkey vultures (0.21 ± 0.33). Both species tended to avoid developed (turkey vultures = − 1.27 ± 2.98; black vultures = − 1.36 ± 3.58) and undeveloped open habitats (turkey vultures = − 0.19 ± 0.34; black vultures = − 0.43 ± 0.40), but turkey vultures consistently selected for developed open areas for diurnal rest sites (0.14 ± 0.63). Both species use of diurnal resting sites proximal and medial to landfills was highest in the warmer months (May – July; turkey vultures = − 0.45 ± 4.53; black vultures = − 0.45 ± 4.52; and turkey vultures = − 0.84 ± 1.74; black vultures = − 0.78 ± 1.71, respectively) and lowest in the cooler months (October – January; turkey vultures = − 2.57 ± 4.73; black vultures = − 2.59 ± 4.62; and turkey vultures = − 3.30 ± 4.41; black vultures = − 3.66 ± 4.53, respectively). Turkey vultures also showed slight selection for areas medial to roads across all months (− 0.05 ± 0.14) compared to black vultures (− 0.24 ± 0.42).

## Discussion

This study provides evidence for niche differentiation in sympatric black and turkey vultures by demonstrating habitat segregation through investigation of spatial data collected at a fine temporal resolution within the southeastern US. Using over 2.8 million locations collected from GPS-tracked black and turkey vultures, our results reveal interspecific differences in monthly roost reuse frequency and monthly resource selection across multiple behavioral states. These data undoubtedly reflect differences in physiology, behavior, and social structure, and thus represent underlying mechanisms of niche differentiation between species. However, alternative mechanisms such as direct competition could influence fine-scale movement behavior and resource use of these species.

Roost reuse and roost site fidelity can be informative parameters differentiating animal behaviors and social structure [48]. For sympatric vultures, we used roost reuse frequency as an index of vulture exploratory behavior and general success in finding new foraging opportunities. As predicted, roost fidelity was lower for turkey vultures than black vultures, as turkey vultures spent more nights in novel roosts than did black vultures. It is expected that turkey vultures would less frequently return to previously used roosts given that they inhabit larger ranges and forage over wider areas than black vultures [8, 9]. Moreover, this finding likely reflects tendencies of black vultures to return to familiar roosts to maintain social connections and communication with conspecifics [16]. These results may have important implications for vulture management and conservation as roost habitat characteristics can be further examined to understand aspects influencing reused and novel roosts.

Although black and turkey vultures have been observed concurrently at evening roosts and diurnal resting sites [38], our data provide clear evidence for niche differentiation across some habitat types and during some months. Throughout all months of the year, turkey vultures selected forested habitats more frequently than black vultures when engaged in flight, diurnal rest, and evening roost behaviors. This finding likely reflects key characteristics of turkey vultures that allow them to utilize resources within forests habitats more efficiently than black vultures. Specifically, turkey vultures have enhanced olfaction [10, 15] and thus typically arrive at carcasses before black vultures [9, 15, 49] and are better able to exploit small carrion in forested areas [50], whereas black vultures must rely more upon visual detection [16] which is limited in forested habitats. Additionally, turkey vultures and black vultures have demonstrated differences in flight abilities influenced by weather conditions and available uplift driven by habitat characteristics [51]. The dihedral wing posture and lower wing loading of turkey vultures allow them to engage in contorted flight more effectively that black vultures [51], particularly when flying low in search of carrion within forested areas. Selection of wooded wetlands by both species for diurnal resting and by turkey vultures for evening roost locations likely reflects the importance of wooded wetlands in providing abundant perch structures year-round and desirable thermal properties [38]. The most apparent difference in selection for roosts within wooded wetlands by species occurred in warmer months (May to July), corresponding with timing of chick-rearing, with turkey vultures increasing their selection of wooded wetlands during this time.

Further distinction in evening roost and diurnal resting habitat selection was evident in use of developed versus undeveloped habitats. While developed/urban, developed/open, and undeveloped/open habitats are all visually unobstructed, facilitating carcass detection, developed/urban and developed/open areas presumably provide more perch structures for resting and roosting than undeveloped/open areas. Selection of diurnal resting sites by turkey vultures in developed open across all months and greater use of undeveloped/open habitats, in particular during March – July, may reflect their enhanced ability to detect and acquire carcasses of smaller-bodied carrion (such as rodents [50]) that are commonly abundant in shrub/scrub and grassland/herbaceous landscapes. However, increased use of roost sites in developed open areas near human activity by black vultures may be higher than that of turkey vultures due to their inherent boldness which can be enhanced in large groups of conspecifics [16, 17]. Although black vultures are primarily obligate scavengers, they have been known to opportunistically attack and kill small livestock, and reports of such depredations are substantial in the southeastern US [52]. Use of roost sites by black vultures in developed/open habitats (including agricultural and livestock production areas) was higher (but still less than was available on the landscape) in April through August, and may reflect springtime livestock activities when calving, lambing, and other livestock births are highest. Turkey vultures also increased selection of flights in developed/open habitats during spring, potentially to take advantage of increased foraging opportunities (e.g., dead livestock, afterbirth) in these habitats during this time. Alternatively, increased use of developed open areas for roosts by black vultures during chick-rearing months may reflect use of abandoned buildings as nesting structures. Further, availability of carrion to black vultures is likely greater in the open terrain of roadways amidst developed urban areas and in fields and agricultural areas because these areas are less visually obstructed than in heavily wooded environments.

Although landfills can provide consistent and reliable forage opportunities, our results suggest vultures may use landfills only in times of greatest need. Use of diurnal resting locations proximal to landfills was highest in warmer months when carrion decomposition rates, and thus competition for ephemeral carrion resources elsewhere, are generally highest. Black and turkey vultures also selected diurnal resting sites closest to landfills similarly in April and May, corresponding with timing of egg-production and incubation for both species. However, we were unable to determine whether any of our vultures were actively breeding over the course of our study and thus it is unknown whether increased use of diurnal resting sites near landfills was in response to young rearing activities or other unrelated factors. Nonetheless, competition for resources among vultures are likely to increase during breeding seasons when energy demands are higher for breeding individuals. Despite occasional daytime selection by both species, proximities nearest to landfills were not strong factors in evening roost site selection for either species in any month. In fact, evening roost locations farthest from landfills (> 5000 m) were utilized more than their proportional availability for both species. A combination of constant disturbance by landfill management activities and low-quality or difficult access to forage resources therein may explain why landfills were not observed to be an important location around which vultures consistently roost in the southeastern US.

Habitat use during flight varied by month and species for nearly all habitat types, although the most notable differences between species occurred in forest and wooded wetlands. Interspecific differences in habitat selection during flight are expected given the different foraging strategies [9] and flight abilities [51] of black and turkey vultures. Turkey vulture flight over forested habitats was substantial throughout the year, while black vulture use of forests varied seasonally, avoiding these areas in cooler months with increasing use during warmer months. Seasonal fluctuations in selection of habitats during flight likely relate to changes in vegetation density throughout the year, with vegetation densities being greatest in the summer and lowest in the winter, particularly in deciduous-dominant wooded wetland stands. Changes in vegetation density can alter the environmental drivers that affect flight conditions (including air temperature and uplift) as well as foraging opportunities [50], particularly for black vultures where visual detection is important. Extensive use of forested habitats during flight by turkey vultures likely reflects their more frequent use of contorted flight as well as enhanced olfaction compared to black vultures and thus ability to locate and exploit carrion under forested canopies [50, 53]. In breeding season, black vultures selected wooded wetlands during flights more than turkey vultures. Given that we also observed substantial use of wooded wetlands by black vultures for diurnal resting and slight selection of wooded wetlands for roosting sites, further suggest the importance of wooded wetlands to black vultures in the southeastern US, particularly during the breeding season.

## Conclusions

Collectively, these data suggest black and turkey vultures exhibit spatial and temporal differences in resource selection, and that these differences are evident in selection of habitats while roosting, resting, and flying. Thus, by evaluating resource selection at a high temporal resolution, and for a range of behaviors, we were able to provide evidence for habitat segregation and niche differentiation for two sympatric species of vultures. These data build upon our understanding of vulture spatial ecology, providing insights into underlying behavioral mechanisms facilitating niche differentiation between species and ultimately should provide critical insights into the conservation and management of this important group of birds. However, alternative mechanisms such as direct competition could influence fine-scale movement behavior and resource use of these species. Future research efforts to examine black and turkey vulture socioecology as well as details on nesting characteristics and food habits (e.g. pellet examination) would further inform management decisions, particularly in areas where these species are considered nuisance.

## Supplementary information

**Additional file 1: Table S1** Habitat variables and associated descriptions for resource selection analyses conducted for black and turkey vultures monitored with GPS transmitters in the southeastern US.

**Additional file 2: Table S2** Comparisons of Akaike Information Criterion (AIC) values of generalized additive mixed models of monthly variation (month) to intercept only models (Int) of selection ratios (sr) for individual habitat types of roosting black and turkey vultures.

**Additional file 3: Table S3**.Comparisons of Akaike Information Criterion (AIC) values of generalized additive mixed models of monthly variation (month) to intercept only models (Int) of selection ratios (sr) for individual habitat types of flying black and turkey vultures.

**Additional file 4: Table S4** Comparisons of Akaike Information Criterion (AIC) values of generalized additive mixed models of monthly variation (month) to intercept only models (Int) of selection ratios (sr) for individual habitat types of resting black and turkey vultures.

## Data Availability

Movement/tracking datasets used in this study are available on Movebank (movebank.org, study name “Black Vultures and Turkey Vultures Southeastern USA”) and are published in the Movebank Data Repository with DOI 10.5441/001/1.67f77j31. Additional datasets generated in support of the conclusions of this article are included within the article and its additional files.

## Abbreviations

AIC: Akaike’s Information Criterion
DNA: Deoxyribonucleic Acid
DOE: Department of Energy
GAMM: Generalized Additive Mixed Model
GIS: Geographic Information System
GPS: Global Positioning System
GSM: Groupe Spécial Mobile
SRS: Savannah River Site
UD: Utilization Distribution
US: United States
VHF: Very High Frequency
