# Supplementary material for: Evidence of niche differentiation for two sympatric vulture species in the Southeastern United States
Source: Mov Ecol. 2019 Oct 30;7:31. doi: 10.1186/s40462-019-0179-z (PMC6822427; doi:10.1186/s40462-019-0179-z)
Supplement: Supplementary file 1 — Additional file 1: Table S1 Habitat variables and associated descriptions for resource selection analyses conducted for black and turkey vultures monitored with GPS transmitters in the southeastern US. [file 40462_2019_179_MOESM1_ESM.pdf]

Table S1. Habitat variables and associated descriptions for resource selection analyses conducted for black and turkey vultures monitored with GPS transmitters in the southeastern U.S.

**Habitat Variable**	**Class\Value from 2011 National Land Cover Database (Homer et al. 2015)**	
Developed/Urban	22	Developed, Low Intensity
	23	Developed, Medium Intensity
	24	Developed, High Intensity
Developed/Open	21	Developed, Open Space
	31	Barren Land (Rock/Sand/Clay)
	81	Pasture/Hay
	82	Cultivated Crops
Undeveloped/Open	52	Shrub/Scrub
	71	Grassland/Herbaceous
Forest	41	Deciduous Forest
	42	Evergreen Forest
	43	Mixed Forest
Wooded Wetland	90	Woody Wetland
Water	11	Water
	95	Emergent Herbaceous Wetland
Landfill (≤50 m)	--	proximal to landfills
Landfill (500-5,000m)	--	medial to landfills
Landfill (>5,000m)	--	distal to landfills
Road (≤500m)	--	proximal to roads
Road (500-5,000m)	--	medial to roads
Road (>5,000m)	--	distal to roads
