# Supplementary material for: Evidence of niche differentiation for two sympatric vulture species in the Southeastern United States
Source: Mov Ecol. 2019 Oct 30;7:31. doi: 10.1186/s40462-019-0179-z (PMC6822427; doi:10.1186/s40462-019-0179-z)
Supplement: Supplementary file 2 — Additional file 2: Table S2 Comparisons of Akaike Information Criterion (AIC) values of generalized additive mixed models of monthly variation (month) to intercept only models (Int) of selection ratios (sr) for individual habitat types of roosting black and turkey vultures. [file 40462_2019_179_MOESM2_ESM.pdf]

Table S2. Model selection results for generalized additive mixed model analyses of vulture roosting locations based on selection ratios (sr) for individual habitat types by species (spp) and month (mo) within 100% home ranges with individual animal (id) as random effect.

		Black Vultures		Turkey Vultures	
**Habitat Type**	**Model**	**AIC**	**Δ**	**AIC**	**Δ**
Forest	s(month)	461.7581	-1.9737	224.35350	-2.0000
	Int	459.7844		222.35350	
Wooded Wetland	s(month)	648.2605	0.9779	161.37450	11.5051
	Int	649.2384		172.87960	
Water	s(month)	744.6374	-1.5933	955.64030	2.4603
	Int	743.0441		958.10060	
Developed/ Urban	s(month)	857.5982	0.9122	949.30300	2.8000
	Int	858.5104		952.10300	
Developed/ Open	s(month)	642.8276	3.0203	789.10440	-2.0000
	Int	645.8479		787.10440	
Undeveloped/ Open	s(month)	698.3188	-1.8095	517.37480	-1.7625
	Int	696.5093		515.61230	
Landfill #1, Distance 0-500m	s(month)	571.8550	-2.0000	736.25690	-2.0000
	Int	569.8550		734.25690	
Landfill #2, Distance >500-5000m	s(month)	716.9660	0.4932	888.93160	3.4903
	Int	717.4592		892.42190	
Landfill #3, Distance >5000m	s(month)	-97.7784	-0.59125	-110.21400	3.6325
	Int	-98.36965		-106.58150	
Road #1, Distance 0-500m	s(month)	856.3063	-1.956	882.25910	-1.3205
	Int	854.3503		880.93860	
Road #2, Distance >500-5000m	s(month)	152.5960	-0.1347	-90.19873	4.6991
	Int	152.4613		-85.49962	
Road #3, Distance >5000m	s(month)	727.9718	5.4516	969.97820	-0.5189
	Int	733.4234		969.45930	
