# Supplementary material for: Evidence of niche differentiation for two sympatric vulture species in the Southeastern United States
Source: Mov Ecol. 2019 Oct 30;7:31. doi: 10.1186/s40462-019-0179-z (PMC6822427; doi:10.1186/s40462-019-0179-z)
Supplement: Supplementary file 3 — Additional file 3: Table S3.Comparisons of Akaike Information Criterion (AIC) values of generalized additive mixed models of monthly variation (month) to intercept only models (Int) of selection ratios (sr) for individual habitat types of flying black and turkey vultures. [file 40462_2019_179_MOESM3_ESM.pdf]

Table S3. Model selection results for generalized additive mixed model analyses of vulture flight locations based on selection ratios (sr) for individual habitat types by species (spp) and month (mo) within 100% home ranges with individual animal (id) as random effect.

		Black Vultures		Turkey Vultures	
**Habitat Type**	**Model**	**AIC**	**Δ**	**AIC**	**Δ**
Forest	s(month)	-36.11788	22.60305	-161.29930	-2.00000
	Int	-13.51483		-163.29930	
Wooded Wetland	s(month)	36.36608	5.5814	-11.73365	2.51618
	Int	41.94748		-9.21747	
Water	s(month)	414.15610	-1.4265	172.14280	0.30980
	Int	412.72960		172.45260	
Developed/ Urban	s(month)	459.16010	-2.0000	359.15410	2.75800
	Int	457.16010		361.91210	
Developed/ Open	s(month)	131.23180	-1.2448	177.31970	11.60140
	Int	129.98700		188.92110	
Undeveloped/ Open	s(month)	-43.46016	-2.0000	-127.34620	0.60020
	Int	-45.46016		-126.74600	
Landfill #1, Distance 0-500m	s(month)	524.47070	5.8225	644.86880	3.29880
	Int	530.29320		648.16760	
Landfill #2, Distance >500-5000m	s(month)	587.61610	14.3963	677.40420	-2.00000
	Int	602.01240		675.40420	
Landfill #3, Distance >5000m	s(month)	-175.97400	-1.6503	-159.75210	5.51230
	Int	-177.62430		-154.23980	
Road #1, Distance 0-500m	s(month)	665.93550	0.1085	418.03080	-1.70000
	Int	666.04400		416.33080	
Road #2, Distance >500-5000m	s(month)	108.61040	24.8282	-227.57580	-0.60870
	Int	133.43860		-228.18450	
Road #3, Distance >5000m	s(month)	603.09220	-2.0000	649.93000	-1.24710
	Int	601.09220		648.68290	
