# Supplementary material for: Evidence of niche differentiation for two sympatric vulture species in the Southeastern United States
Source: Mov Ecol. 2019 Oct 30;7:31. doi: 10.1186/s40462-019-0179-z (PMC6822427; doi:10.1186/s40462-019-0179-z)
Supplement: Supplementary file 4 — Additional file 4: Table S4 Comparisons of Akaike Information Criterion (AIC) values of generalized additive mixed models of monthly variation (month) to intercept only models (Int) of selection ratios (sr) for individual habitat types of resting black and turkey vultures. [file 40462_2019_179_MOESM4_ESM.pdf]

Table S4. Model selection results for generalized additive mixed model analyses of vulture diurnal resting locations based on selection ratios (sr) for individual habitat types by species (spp) and month (mo) within 100% home ranges with individual animal (id) as random effect.

		Black Vultures		Turkey Vultures	
**Habitat Type**	**Model**	**AIC**	**Δ**	**AIC**	**Δ**
Forest	s(month)	165.13520	15.83140	63.43684	14.85155
	Int	180.96660		78.28839	
Wooded Wetland	s(month)	173.03490	-1.90150	93.59149	4.77005
	Int	171.13340		98.36154	
Water	s(month)	721.38630	-1.63670	519.80700	12.05400
	Int	719.74960		531.86100	
Developed/ Urban	s(month)	811.12040	-0.42820	833.74180	-2.00000
	Int	810.69220		831.74180	
Developed/ Open	s(month)	437.43500	8.45880	283.28840	1.21990
	Int	445.89380		284.50830	
Undeveloped/ Open	s(month)	147.53630	-0.86800	97.92261	8.31190
	Int	146.66830		106.23451	
Landfill #1, Distance 0-500m	s(month)	615.33310	3.15600	749.79990	10.76630
	Int	618.48910		760.56620	
Landfill #2, Distance >500-5000m	s(month)	643.48920	11.95860	797.25620	4.71290
	Int	655.44780		801.96910	
Landfill #3, Distance >5000m	s(month)	-188.82640	0.81940	-123.68320	3.68820
	Int	-188.00700		-119.99500	
Road #1, Distance 0-500m	s(month)	791.27840	5.04400	641.79630	-0.28830
	Int	796.32240		641.50800	
Road #2, Distance >500-5000m	s(month)	57.91426	5.45432	-193.71420	3.30370
	Int	63.36858		-190.41050	
Road #3, Distance >5000m	s(month)	721.96950	1.19010	883.38670	6.84290
	Int	723.15960		890.22960	
